# Patient-reported outcome after stemmed versus stemless total shoulder arthroplasty for glenohumeral osteoarthritis: a patient-blinded randomized clinical trial

**DOI:** 10.1186/s13063-019-3535-9

**Published:** 2019-07-12

**Authors:** Zaid Issa, Jeppe Vejlgaard Rasmussen, John Kloth Petersen, Kim Schantz, Stig Brorson

**Affiliations:** 1 Department of Orthopedic Surgery, Zealand University Hospital, University of Copenhagen, Koege, Denmark; 2 Department of Orthopedic Surgery, Herlev, Gentofte Hospital, University of Copenhagen, Herlev, Denmark; 3 Department Of Orthopedic Surgery, Hvidovre Hospital, University of Copenhagen, Hvidovre, Denmark

**Keywords:** Shoulder arthroplasty stemless, Non-stemmed, Shoulder prosthesis, Shoulder replacement, Shoulder osteoarthritis, Metaphyseal fixation

## Abstract

**Background:**

Stemless shoulder arthroplasty systems with uncemented metaphyseal fixation have been used for glenohumeral osteoarthritis since 2004 (Hawi, et al. BMC Musculoskelet Disord 17:376, 2016). The stemless design has several theoretical advantages compared with the stemmed shoulder arthroplasty systems: restoring patients’ anatomy; preserving humeral bone stock; and few complications in component removal if the need for a revision arthroplasty arises. The purpose of the study is to compare the short-term, patient-reported outcome of stemless and stemmed total shoulder arthroplasty (TSA).

**Materials and methods:**

A randomized clinical trial will be conducted. Eighty patients with clinical and radiological signs of primary or post-traumatic glenohumeral osteoarthritis, computed tomography (CT) scan-verified adequate glenoid bone stock, and no total rupture of rotator cuff tendons verified by a magnetic resonance imaging (MRI) scan will be randomly allocated to a stemless or stemmed TSA. The primary outcome will be the Western Ontario Osteoarthritis Shoulder (WOOS) score at 12 months. Secondary outcomes are the WOOS score at three months and the Oxford Shoulder Score (OSS) and EQ-5D at 3 and 12 months. All complications, including glenoid and humeral component loosening, instability, rotator cuff tear, intraoperative and postoperative periprosthetic fracture, nerve injury, infection, deltoid injury, and symptomatic deep venous thrombosis, will be reported.

**Discussion:**

Findings will provide patients with better information about the potential benefits and harms of stemless and stemmed TSA and will assist shoulder surgeons and patients in decision-making.

**Trial registration:**

Clinicaltrials.gov, NCT03877315. Registered on 13 March 2019.

**Electronic supplementary material:**

The online version of this article (10.1186/s13063-019-3535-9) contains supplementary material, which is available to authorized users.

## Background

Glenohumeral joint osteoarthritis is a common cause of shoulder pain, affecting up to one-third of patients aged > 60 years [1]. Surgical treatment is indicated for patients with glenohumeral arthritis who continue to experience significant symptoms despite an appropriate course of non-operative management. Shoulder arthroplasty accounts for the third most common joint replacement procedure after hip and knee arthroplasty [2]. Based on data from the National Patient Registry, the Statistical Department of the Danish National Board of Health, the use of primary shoulder replacement in Denmark increased from 12 replacements per 100,000 inhabitants in 2005 to 19 replacements per 100,000 inhabitants in 2015 [3]. Glenohumeral osteoarthritis is now the most common indication for total shoulder arthroplasty (TSA) in Denmark [3].

Many different TSA designs are available on the market. The stemless shoulder arthroplasty system with uncemented metaphyseal fixation has been used in Europe for glenohumeral osteoarthritis since 2004. The indications for anatomical stemless TSA are the same as for anatomical stemmed TSA: osteoarthritis; rheumatoid arthritis; and post-traumatic osteoarthritis or osteonecrosis.

The contraindications for anatomical stemless TSA are acute proximal humerus fracture, inadequate metaphyseal bone stock, and rotator cuff insufficiency [4, 5].

Stemless TSA has several theoretical advantages over stemmed TSA: restoring patients’ anatomy (humeral shaft angle, humeral head diameter, and lateralization); preserving humeral bone stock; and few complications in component removal should the need of a revision arthroplasty arise [6–8]. A recent review of 3360 anatomical TSAs found an overall complication rate of 10.3% [8]. Periprosthetic fractures accounted for 6.7% and humeral component loosening for 1.4% of all complications. Complications related to the stemmed humeral component can be divided into intraoperative (malpositioning, false route, periprosthetic fracture) and postoperative (loosening, migration, disassembly, periprosthetic fracture, stem fracture) complications [9]. When a revision is necessary because of infection or periprosthetic fracture, the removal of a well-fixed or cemented humeral component can be challenging and lead to bone damage [10].

Few outcome studies on stemless TSA are available. A recent review of 11 observational studies (published 2010–2016), incorporating a total of 929 patients, reported comparable short- and mid-term functional outcomes between stemmed and stemless shoulder prosthesis [11]. Otherwise, there are few well-conducted and adequately powered clinical studies.

The objective of this study is to increase knowledge about shoulder function after operation with anatomical TSA by comparing the patient-reported outcome after stemmed and stemless anatomical TSA for glenohumeral osteoarthritis.

The Standard Protocol Items Recommendations for Interventional Trials (SPIRIT) Statement 2013 has been followed for the completion of this protocol (Table 1 and Additional file 1).

**Table 1:**
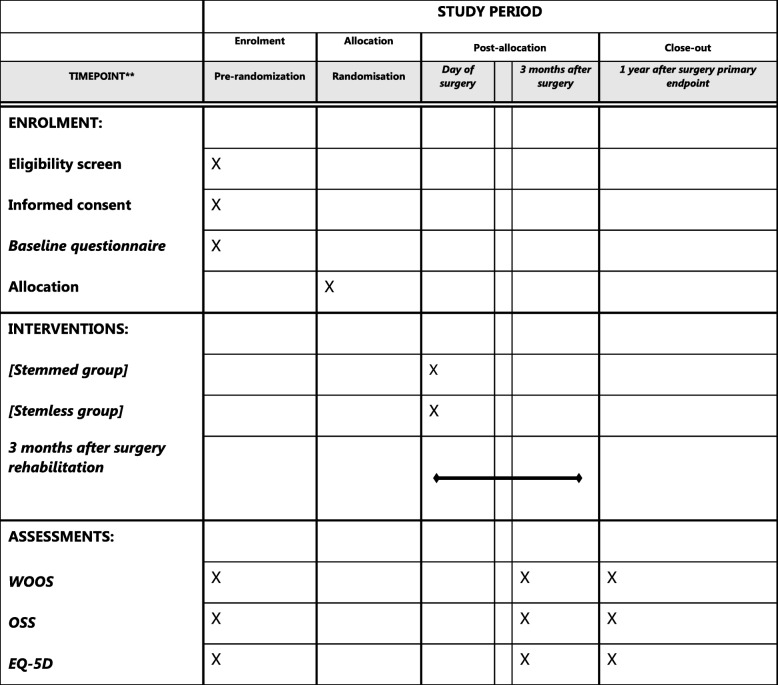
Standard Protocol Items Recommendations for Interventional Trials (SPIRIT) diagram

*Stemmed group* patients operated with stemmed shoulder arthroplasty, Biomet Comprehensive® Total Shoulder System, *Stemless group* patients operated with stemless shoulder arthroplasty, Biomet Comprehensive® Nano Shoulder System *WOOS* Western Ontario Osteoarthritis Shoulder, *OSS* Oxford Shoulder Score, *EQ-5D* the health status component of the EuroQol assessment

## Methods/Design

### Study design

An investigator-initiated, blinded (patient and data analyzer), non-inferiority, randomized controlled clinical trial.

### Material

**Fig. 1:**
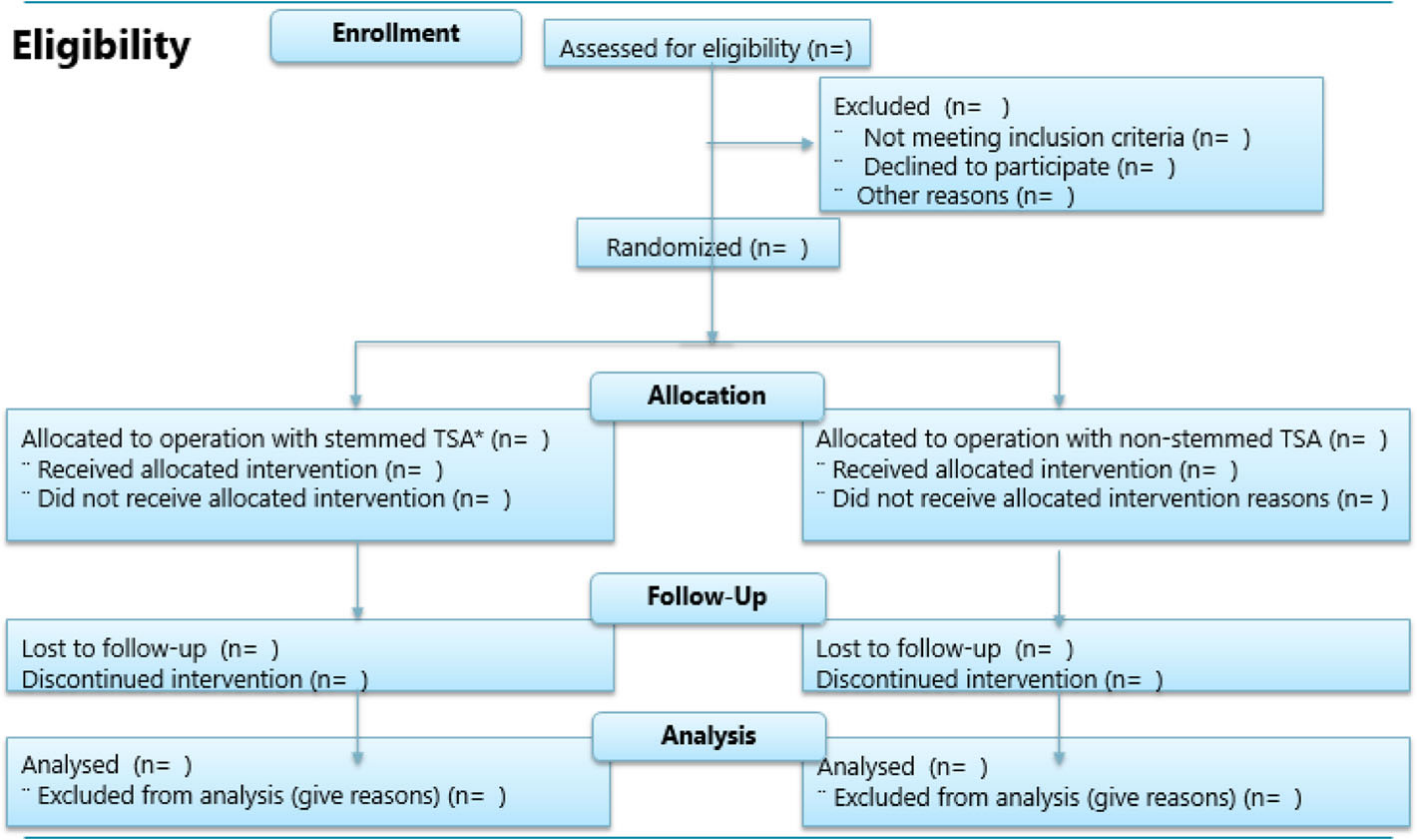
CONSORT 2010 *flow diagram*. The expected flow of patients through the study. TSA total shoulder arthroplasty

Seventy-eight patients with shoulder osteoarthritis randomized to either stemmed or stemless shoulder prosthesis. The flow of participants into the study are demonstrated in Fig. 1

### Inclusion criteria

1. Patients aged ≥ 18 years.
2. Patients have been referred to the Orthopedic Department or Zealand University Hospital, Koege or Hvidovre University Hospital for shoulder pain during the study period.
3. Patients have the ability to read trial information in Danish and give informed consent.
4. The diagnosis will be based on plain radiographs (at least two perpendicular views), reduction of joint space, and/or osteophyte formation.
5. Clinical presentation with pain at night and/or daily pain, pain in overhead activity, and consumption of pain medication.

### Exclusion criteria

1. Previous shoulder surgery that involves the humeral head and/or the glenoid cavity.
2. Patients with alcohol or drug abuse problems that can compromise rehabilitation and follow-up appointments as assessed by the recruiting surgeon at the first visit.
3. Patients unable to understand instructions in Danish, follow the rehabilitation protocol, or answer the questionnaires because of physical or cognitive inabilities as evaluated by the recruiting surgeon at the first visit.
4. Brachial plexus palsy.
5. Patients with previous fractures around the shoulder (clavicle, scapula, and proximal humerus fractures).
6. Patients with magnetic resonance imaging (MRI) scan-verified full thickness total tear of one or more of the rotator cuff tendons.
7. Patients with computed tomography (CT) scan-verified glenoid retroversion ≥ 20° that does not allow glenoid component fixation without bone graft or need an augmented glenoid component [12].

### Eligibility

Patients referred with the diagnosis of “glenohumeral osteoarthritis” will be examined by clinical examination and plain radiographs to confirm the diagnosis. In the case of glenohumeral osteoarthritis, which is to be treated with TSA, the shoulder surgeon will offer the patient participation in the trial at the first visit; the patient will be referred to routine shoulder CT and MRI scans (which are already a part of standard shoulder osteoarthritis protocol) and receive a folder with patient participant information and “Researchers rights in a health science research project.”

Patients will have time to consider participation before the next visit (approximately one month) after the first visit.

At the second visit, if the patients give their informed consent for participation in the study, they will receive three questionnaires to fill out while undisturbed in a room next door before handing them to a secretary who will check that the questionnaires are completed before the patient leaves the hospital.

The informed consent gives the primary investigator access to information about the patient’s health condition from the medical record, including data on age, sex, American Society of Anesthesiologists (ASA) score, previous shoulder operations, and results from shoulder CT and MRI scans.

All surgical interventions will be undertaken at the Zealand University Hospital Koege and Hvidovre Hospital. A two-year recruitment period from 01 March 2019 to 01 January 2022 is planned.

### Randomization and concealment of allocation

A computerized irreversible randomization application, Research Electronic Data Capture (REDCap)© [13], will allocate patients into two equal groups:

- Operated with stemmed shoulder arthroplasty, Biomet Comprehensive® Total Shoulder System; and
- Operated with stemless shoulder arthroplasty, Biomet Comprehensive® Nano Shoulder System.

The randomization sequence will be computer-generated by block randomization (four in each block) and balanced according to age in years (18–49, 50–59, 60–69, 70–79, 80–89, 90–110), sex, and ASA score. The randomization (patient allocation) will be performed on the trial laptop computer in the operation room while the patient is being anesthetized in the preparation room next door. The operating surgeon will obtain the allocation code by logging onto the REDCap website after positioning the anesthetized patient in a beach chair position. After randomization, the patient’s allocation will be revealed to the operation team to unpack the necessary operation tools and prosthesis.

### Blinding

Patients will be blinded to which implant type they are to receive. This will be done by not sharing the operation chart files with the patient’s online file records; the inserted shoulder prosthesis will be noted by a unique code in operation file, and the radiologist will be asked not to describe the postoperative X-ray controls.

The primary investigator will conduct the blinded statistical analysis supervised by the biostatistician.

### Interventions

The operations will be performed by four shoulder surgeons familiar with the procedures of both shoulder arthroplasty systems. All patients will be operated on with the deltopectoral approach with subscapularis and biceps tenodesis and instrumentation as described by the manufacture [14].

All patients will receive standard pre- and perioperative pain management, including general anesthesia and interscalene peripheral nerve block performed by the anesthesiologist.

Postoperative pain management will be adjusted regarding each patient’s needs and recorded. We do not expect that individualized pain management will affect our primary outcome measure one year after surgery.

### Physiotherapy and self-training

All patients will receive a standardized rehabilitation program at their local municipality service. The physiotherapist in the hospital will demonstrate training protocol and instructions the day after surgery.

From 0 to 6 weeks, postoperative passive-to-active unloaded movements in all directions are allowed, with the following exceptions:

1. No external rotation is allowed in the first three weeks. After three weeks, external rotation is allowed until the feeling of capsular tightening;
2. No internal rotation of the operated arm behind the back is allowed in the first six weeks.

## Outcome measures

The primary outcome will be the Western Ontario Osteoarthritis Shoulder (WOOS) score at 12 months [15].

The secondary outcomes will measure the following:

1. WOOS score at three months;
2. OSS at 3 and 12 months postoperatively [16];
3. General health status questionnaire EQ-5D at 3 and 12 months [17].

At baseline, three-, and twelve-month visits, patients will fill out questionnaires, WOOS, OSS, and EQ-5D, alone before leaving the hospital (at the second visit after accepting participation) and in the waiting room (at three- and twelve-month visits) without interference from the medical staff.

### Follow-up

To ensure that the prosthesis is in situ, all patients will undergo a conventional X-ray in two perpendicular views before discharge. The discharge will be on day 1 or day 2 postoperatively, depending on the patient’s pain and the need for recruiting social help service at home.

All patients will be followed actively for one year postoperatively. Further follow-up including revision rates will be recorded at five and ten years after surgery using data from the Danish Shoulder Register (DSR).

Complications and adverse events that may have developed after the 12-month evaluation but that did not lead to revision surgery will be extracted from the patient records (e.g. discharge letters, discharge diagnosis, needed journal notes).

## Outcome assessment tools

### Western Ontario Osteoarthritis of the Shoulder Score (WOOS)

The WOOS is a patient-administered, disease-specific questionnaire for the measurement of the quality of life of patients with osteoarthritis. It provides scores for four domains: (1) physical symptoms; (2) sport, recreation, and work; (3) lifestyle; and (4) emotions. Patients answer each question using the visual analogue scale. The WOOS score is calculated by measuring the distance from the left side of the line and calculating the possible score in the range of 0–100 (recorded to the nearest 0.5 mm.) It consists of 19 questions; the total score is in the range of 0–1900. A maximum score of 1900 signifies that the patient has an extreme decrease in shoulder-related quality of life, whereas a score of 0 signifies that the patient has no decrease in shoulder-related quality of life. The raw scores are converted to a percentage of the maximum score for simplicity of presentation. The questionnaire has been translated into Danish and validated and tested on patients with shoulder osteoarthritis [15, 18, 19].

### Oxford Shoulder Score (OSS)

The OSS is a measurement tool for the assessment of outcomes of shoulder surgery [16, 20]. It has been tested and validated in patients with primary or secondary osteoarthritis. The OSS is a 12-item questionnaire, with each item scored in the range of 0–4; thus, the overall score is the sum of the scores received for individual questions. This results in a continuous score ranging from 0 (most severe symptoms) to 48 (least severe symptoms) [16]. For simplicity of presentation, the raw scores will be converted to a percentage of the maximum score. We will use a validated Danish version [20].

### EQ-5D

The EQ-5D, the health status component of the EuroQol assessment (EuroQol Group, Rotterdam, The Netherlands), is a generic instrument for describing and evaluating health-related quality of life. The EuroQol instrument has been designed for self-completion by the respondent.

The EQ-5D is a descriptive system comprising five dimensions in each of which the respondents describe their health state: mobility; self-care; usual activities; pain/discomfort; and anxiety/depression. Each dimension has five levels: no problems; slight problems; moderate problems; severe problems; and extreme problems. The patients are asked to indicate their health state by ticking the box next to the most appropriate statement in each of the five dimensions, which corresponds to a one-digit number that expresses the level selected for that dimension. The patient chooses one of five levels for each dimension; thus, a five-digit number (EuroQol Group 1990) can define the resulting health state. The reliability and validity of the EQ-5D have been evaluated in different patient populations, including the Danish population, with the conclusion that the assessment had good validity, reliability, and responsiveness [17, 21]. The EQ-5D demonstrated good internal and external responsiveness in patients with shoulder injuries and can therefore be used as an outcome measure for evaluating the HRQOL in both clinical studies and healthcare assessments [22–24]. We received permission to use the Danish version (permission ID number 27296) [25].

### Patient dropout and protocol violations

Patients who drop out of the trial will be recorded and the reason for dropout will be noted. The principal investigator will record and report any protocol violations.

### Side effects and adverse events reporting

The department routinely uses both types of shoulder prostheses. The treatments’ risks and disadvantages are therefore not expected to differ from normal patient application outside the trial. They include superficial infection, deep infection, loosening of the prosthesis, nerve or vascular injury during surgery, lack of healing, and prosthesis migration. The amount of ionizing radiation to which the patients are exposed during the CT scan and X-rays does not differ from that for patients outside the study.

To assess possible complications after prosthesis insertion, we will record any case of deep infection, nerve injury, implant loosening (based on radiographic images), heterotopic ossification, instability, dislocation, implant removal, and revision surgery or any adverse event that leads to hospitalization one year after surgery. All complications will be reviewed at the end of the trial and reported.

## Statistical analysis plan

### Sample size and power calculation

No existing studies report the minimal clinically important difference (MCID) of WOOS, but the MCID of the OSS was investigated and found to be 6 points or 12.5% of a maximum score [26]. By extrapolating MICD from OSS to WOOS, based on the known MCID for OSS, we estimated the accepted clinically meaningful change (ACMC) of WOOS to 237.5 points (12.5%).

With an ACMC of 12.5% and a standard deviation (SD) of ±20.0, which we found from our retrospective evaluation of patients operated with stemless TSA in our department, we need to include 64 patients, 32 in each group, to be 80% sure that the lower limit of a one-sided 95% confidence interval will be above the non-inferiority threshold of – 12.5%. With a loss of follow-up assumption of 20%, a total of 78 patients is required [27].

### Hypotheses

We hypothesize the following:

- A one-year postoperative WOOS score for patients operated with stemless TSA is inferior to that of patients operated with stemmed TSA by at least ACMC = 237.5 (12.5%).

$$ \left({\mathrm{H}}_0:{\upmu}_{\mathrm{stemmed}}>{\upmu}_{\mathrm{stemless}}+\mathrm{ACMC}\right) $$

- A one-year postoperative WOOS score for patients operated with stemless TSA is not inferior to that of patients operated with stemmed TSA by ACMC = 237.5 (12.5%).

$$ \left({\mathrm{H}}_{\mathrm{A}}:{\upmu}_{\mathrm{stemmed}}\le {\upmu}_{\mathrm{stemless}}+\mathrm{ACMC}\right) $$

#### Data analysis

No interim data analysis will be carried out. The primary investigator will enter data in the REDCap database, and the principle investigator before the data analysis will perform proofreading of a random sample of 20 patients.

Data analysis will be conducted by a blinded biostatistician. Patients who drop out after the three-month evaluation will be included in the analysis based on three months of data (“last observation carried forward”). Patient dropouts before the three months’ evaluation will not be included in the analysis unless they participate in the one-year questionnaire evaluation. As suggested in the extension of the CONSORT statement for non-inferiority trial design, a per-protocol analysis will be conducted and results obtained [28, 29]. A questionnaire containing one or more missing answers will be marked as incomplete; the patients will be contacted by letter to answer the missing questions.

Descriptive statistics will be used to report demographic data. Differences in demographic data and outcome measures between the two groups will be compared using chi-square test for categorical variables; parametric (Student’s t-test) or non-parametric (Mann–Whitney U-test) test for continuous variables depending on the nature of data. Data will be checked for possible extreme values before the analysis. The statistical analyses will be performed using SPSS version 25.0 (IBM Corp, Armonk, NY, USA) [30]. The level of statistical significance is set at *P* < 0.05; all *P* values are two-tailed. All complications, including glenoid and humeral component loosening, glenoid wear instability, rotator cuff tear, intraoperative and postoperative periprosthetic fracture, nerve injury, infection, deltoid injury, and deep venous thrombosis will be reported. Our sample size does not give us enough power to allow comparison.

## Discussion

Through randomization and blinding, we aim to decrease the risk of bias. The use of usual care increases the study’s validity and the results can be generalized for the rest of this patient population. The current study will provide high-quality evidence regarding the short-term patient-reported outcome of stemless and stemmed TSA. We will attempt to avoid the type-II error (when hypothesis testing does not reject the null hypothesis, even though the null should have been rejected) by using a proper sample and by adding 20% to the sample size compensating for dropouts. However, we will reduce dropout bias by obtaining permission (at the first visit) to call patients who do not show up for subsequent visits. We will avoid selection bias through randomization and reduce interviewer bias by letting patients answer the questionnaires without interference from the medical staff and hand it back to the receptionist, who will check only that all the questionnaires are filled out. Misclassification of non-differential information bias of the outcome can affect the results in both groups, but errors in outcome classification tend to be less common and have much less impact on the estimate of association. It tends to minimize any true difference between the groups (bias toward the null). Using stemless rather than stemmed prosthesis regarding revision surgery might have some advantages, but this study design does not allow us to investigate potential advantages or disadvantages; further studies must be carried out to address such issues.

### Trial status

Protocol version 3.

Issue date: 22 January 2019, Author ZI.

Recruitment was initiated on 15 April 2019 and is expected to be finalized by 1 April 2022. (In case the target number of 78 patients has not been met, the recruitment period may be extended to reach the number required.)

The trial was registered on 13 March 2019 in clinicaltrials.gov (NCT03877315).

## Additional files

Additional file 1: SPIRIT 2013 Checklist: Recommended items to address in a clinical trial protocol and related documents* (DOC 122 kb)

Additional file 2: The Scientific Ethics Committee in Region Zealand, Denmark approvement this study protocol. (PDF 363 kb)

Additional file 3: Translated funding letter has obtained from the research foundation for health research of The Zealand Region (18–000494) for the principal investigator’s (ZI) salary during research periods. (DOCX 13 kb)

## Data Availability

All study-related information on participants will be stored securely on the study site. All completed paper forms will be stored in locked file cabinets before and after data are entered in the electronic database. Study data will be collected and managed using REDCap electronic data capture tools hosted in the Capital Region of Denmark [13]. REDCap is a secure, web-based application designed to support data capture for research studies. All electronic participants’ information will be stored on a secured study-specific drive at the study site. Access to the study-specific drive will be limited to users currently working on the project and will be restricted by individual person-specific login and passwords. The primary investigator will keep an updated list of persons with access to the study-specific drive. Appointment schedules and any other listings linking participants to additional identifying information will be stored separately in the hospital’s secured intranet system or separate locked file cabinets in an area with limited access. Study information will not be released outside of the study without the permission of the relevant participant. The study is being reported to and approved by the Danish Data Protection Agency and adheres to the Act on Processing Personal Data.

## Abbreviations

ACMC: Accepted clinically meaningful change
ASA: American Society of Anesthesiologists Score
CT: Computed tomography
DSR: Danish Shoulder Arthroplasty Register
MCID: Minimal clinically important differences
MRI: Magnetic resonance imaging
OSS: Oxford Shoulder Score
TSA: Total shoulder arthroplasty
WOOS: Western Ontario Osteoarthritis Shoulder
