# Supplementary material for: Patient-reported outcome after stemmed versus stemless total shoulder arthroplasty for glenohumeral osteoarthritis: a patient-blinded randomized clinical trial
Source: Trials. 2019 Jul 12;20:427. doi: 10.1186/s13063-019-3535-9 (PMC6626414; doi:10.1186/s13063-019-3535-9)
Supplement: Supplementary file 2 — The Scientific Ethics Committee in Region Zealand, Denmark approvement this study protocol. (PDF 363 kb) [file 13063_2019_3535_MOESM2_ESM.pdf]

English translation of ethical approval letter.

Committee approval of SJ-750 "Patient-reported outcome after total shoulder joint prosthesis with stemmed vs. stemless shoulder arthroplasty for treatment of osteoarthritis in the shoulder: A randomized, patient-blinded clinical trial."
The Scientific Ethics Committee for Region Zealand has considered the notified research project at its meeting on 6 February 2019.

Committee's decision
The committee has approved the project in accordance with Act No. 593 of 14 June 2011 on scientific ethical treatment of health science research projects (the Committee Act).
The approval of the project is valid until 31 December 2022 and includes the following documents:

- Protocol dated 22 January 2019, version 3, received by mail of 22 January 2019 and the questionnaires related to the protocol
- Participant information dated 22 January 2019, version 3, received in the mail of 22 January 2019
- Declaration of consent dated 15 January 2019, version 2, received by mail of 17 January 2019

The approval applies to the notified test sites and the notified experimental manager in Denmark.
It should be noted that the Committee is not the authority of the Data Protection Code.
The Committee assumes that the project is implemented and the information provided to the participants is in accordance with the Data Protection Regulation and the Data Protection Act.
Please note that the Data Protection Officer is responsible for notifying the data subject that information is being processed.
The Data Protection Regulation contains a number of requirements for, inter alia, content, and form of notification. Read more about the information obligation (Articles 13 and 14) and about the other persons' rights in the Data Inspectorate's guidance on the rights of the data subjects (see www.datatilsynet.dk).
Date: February 19, 2019
Widely: 3904503 Secretariat of the Scientific Ethics Committee Region Zealand
Alléen 15 4180 Sorø
Tel: 57 87 52 83
Rvk-sjaelland@regionsjaelland.dk
www.regionsjaelland.dk

Changes
If significant changes are made to the protocol material during the implementation of the project, these must be notified to the committee in the form of additional protocols. The amendments must only be implemented after approval by the committee, cf. First
Notification of additional protocols must be made electronically via the committee's website, where there is a link to the notification form, and with the use of notification number: 66271.
Important changes include changes that may affect the safety of the subjects, the interpretation of the scientific documentation on which the project is based, and the implementation or management of the project. It can be changing in inclusion - and exclusion criteria, experimental design, number of subjects, test procedures, duration of treatment, effect parameters, changes in the experimental or experimental sites, and content changes in the written information material for the subjects.
Where new information means that the researcher is considering changing the procedure or stopping the experiment, the committee must be informed.

Side effects and events
Continuous reporting
The committee must be informed immediately if, during the project, serious, unexpected side effects or serious incidents are suspected, cf. 1. The report must be accompanied by comments on any consequences for the trial. Only side effects and incidents have occurred in Denmark that must be reported. Notification must be made no later than 7 days after the sponsor or the trial manager has been informed of the case.
When reporting, a form can be used, which can be found on the National Scientific Ethics Committee's (NVK) website. The form with appendices can be submitted electronically using a digital signature to RVK-Sjaelland@regionsjaelland.dk.

Annual reporting
Once a year throughout the trial period, the committee must have sent a list of all suspected serious (expected and unexpected) side effects and serious events that occurred during the trial period together with a report on the safety of the subjects, cf. 2. The material must be in Danish or English. When reporting, a form must be used, which can be found on the NVK's website. The form with bi-layers can be submitted electronically using a digital signature to RVK-Sjaelland@region-sjaelland.dk.

Ending
The experimental officer must notify the committee within 90 days of the completion of the project, cf. 1. The project is considered complete when the last subject is completed. When submitting the report, a form must be used which can be found on the NVK's website.
If the project is interrupted earlier than planned, a reason for this must be sent to the committee no later than 15 days after the decision has been made, cf. 2nd
If the project does not start, this must be communicated to the committee together with a justification.
The committee asks for a copy of the final research report or publication, cf. 2. In this connection, we must point out that there is a duty to publish both negative, positive and inclusive trial results, cf. 1, no. 8.
Oversight
The committee supervises that the project is carried out in accordance with the approval, cf. section 28 and section 29 of the Committee Act.
The committee participated in the proceedings

- Ellen Astrid Holm
- Ole Birger Vestager Pedersen
- Gitte Rinds Andersen
- Annemarie Knigge
- Egon Bo
- Kirsten-Marie Devantier
- Charlotte Petersson
- Bodil Lake

Yours sincerely
Tanja Schwartzbach Frederiksen

Translation performed by the primary investigator Zaid Issa and approved by his principal superviser Stig Brorson.

10-03-2019
