# Supplementary material for: Patient-reported outcome after stemmed versus stemless total shoulder arthroplasty for glenohumeral osteoarthritis: a patient-blinded randomized clinical trial
Source: Trials. 2019 Jul 12;20:427. doi: 10.1186/s13063-019-3535-9 (PMC6626414; doi:10.1186/s13063-019-3535-9)
Supplement: Supplementary file 3 — Translated funding letter has obtained from the research foundation for health research of The Zealand Region (18–000494) for the principal investigator’s (ZI) salary during research periods. (DOCX 13 kb) [file 13063_2019_3535_MOESM3_ESM.docx]

**English translation of funding letter/e-mail**

From: Region SJ <bobm@regionsjaelland.dk>

Date: 26/02/2019 14.17 (GMT + 01: 00)

To: Stig Brorson <sbror@regionsjaelland.dk>

Subject: Region Zealand Health Sciences Research Fund 2018/2019

Dear Stig Brorson

Application for research funding from Region Zealand's Health Sciences Research Fund (RSSF) 2018/2019:

Project title: Clinical outcome after stemless shoulder arthroplasty for glenohumeral osteoarthritis

Application number: R17A66B14

The RSSF Fund Board has processed your application and has decided to support the project with DKK 341.695

The grant may only be used for the project above and the purpose for which it was requested.

The grant will be transferred to the hospital at the upcoming budget transfer on March 31, 2019, and administered by the hospital's finance department. You should, therefore, contact the hospital's finance office within 14 days and inform them about your grant. The finance office will make sure to create a project account for your project under your department's budget. The costs of the project must be endorsed by your department management before submitting it to the accounting office.

Since the grant will be added to your department, it will also be your department, who must ensure that any transfer is made before the end of the year. Unused grant for next year's budget.

If there are excess funds from the grant, you must contact Data and development support by e-mail: reg.forsk@regionsjaelland.dk for the purpose of reversing the balance.

The Health Sciences Research Fund comes in new grants for 2020 with the deadline for applications early September 2019 and is announced at: https://www.regionsjaelland.dk/Health/research/forfagfolk/forskningsfinansiering/Sider/oekonomi.aspx

*Translation performed by the primary investigator Zaid Issa and approved by his principal superviser Stig Brorson.*

*10-03-2019*
